# Supplementary material for: An open-source framework for large-scale, flexible evaluation of biomedical text mining systems
Source: J Biomed Discov Collab. 2008 Jan 29;3:1. doi: 10.1186/1747-5333-3-1 (PMC2276192; doi:10.1186/1747-5333-3-1)
Supplement: Additional file 1 — Evaluation platform source code. The additional file contains the software evaluation framework discussed in this paper. The most current release can be downloaded from SourceForge.net [1]. See the accompanying README file for instructions on installation and use. Also included in the distribution is a collection of UIMA wrappers for some commonly used BioNLP tools and annotated corpora. [file 1747-5333-3-1-S1.zip › bionlp_uima_repository-1.0.1/docs/api/src-html/edu/uchsc/ccp/uima/mention/CCPClassMention.html]

```
001    
002    
003    /* First created by JCasGen Tue Aug 28 16:44:10 MDT 2007 */
004    package edu.uchsc.ccp.uima.mention;
005    
006    import org.apache.uima.jcas.JCas;
007    import org.apache.uima.jcas.JCasRegistry;
008    import org.apache.uima.jcas.cas.FSArray;
009    import org.apache.uima.jcas.cas.TOP_Type;
010    
011    import edu.uchsc.ccp.uima.annotation.CCPTextAnnotation;
012    
013    
014    /** The CCP ClassMention is the root of a flexible class structure that can be used to store virtually any frame-based representation of a particular class. Common class mention types include, but are not limited to, such things as entities (protein, cell type, cell line, disease, tissue, etc.) and frames (interaction, transport, regulation, etc.).
015     * Updated by JCasGen Tue Aug 28 16:44:10 MDT 2007
016     * XML source: /home/williamb/eclipse/RELEASED_SOURCE_CODE/descriptors/typeSystem/CCPTypeSystem.xml
017     * @generated */
018    public class CCPClassMention extends CCPMention {
019      /** @generated
020       * @ordered 
021       */
022      public final static int typeIndexID = JCasRegistry.register(CCPClassMention.class);
023      /** @generated
024       * @ordered 
025       */
026      public final static int type = typeIndexID;
027      /** @generated  */
028      public              int getTypeIndexID() {return typeIndexID;}
029     
030      /** Never called.  Disable default constructor
031       * @generated */
032      protected CCPClassMention() {}
033        
034      /** Internal - constructor used by generator 
035       * @generated */
036      public CCPClassMention(int addr, TOP_Type type) {
037        super(addr, type);
038        readObject();
039      }
040      
041      /** @generated */
042      public CCPClassMention(JCas jcas) {
043        super(jcas);
044        readObject();   
045      } 
046    
047      /** <!-- begin-user-doc -->
048        * Write your own initialization here
049        * <!-- end-user-doc -->
050      @generated modifiable */
051      private void readObject() {}
052         
053     
054        
055      //*--------------*
056      //* Feature: slotMentions
057    
058      /** getter for slotMentions - gets A class mention optionally has slot mentions which represent attributes of that class. These slot mentions are stored in the slotMentions FSArray. There are two types of slot mentions, complex and non-complex.  The difference between complex and non-complex slot mentions is simply the type of filler (or slot value) for each. Complex slot mentions are filled with a class mention, whereas non-complex slot mentions are filled by simple Strings.
059       * @generated */
060      public FSArray getSlotMentions() {
061        if (CCPClassMention_Type.featOkTst && ((CCPClassMention_Type)jcasType).casFeat_slotMentions == null)
062          jcasType.jcas.throwFeatMissing("slotMentions", "edu.uchsc.ccp.uima.mention.CCPClassMention");
063        return (FSArray)(jcasType.ll_cas.ll_getFSForRef(jcasType.ll_cas.ll_getRefValue(addr, ((CCPClassMention_Type)jcasType).casFeatCode_slotMentions)));}
064        
065      /** setter for slotMentions - sets A class mention optionally has slot mentions which represent attributes of that class. These slot mentions are stored in the slotMentions FSArray. There are two types of slot mentions, complex and non-complex.  The difference between complex and non-complex slot mentions is simply the type of filler (or slot value) for each. Complex slot mentions are filled with a class mention, whereas non-complex slot mentions are filled by simple Strings. 
066       * @generated */
067      public void setSlotMentions(FSArray v) {
068        if (CCPClassMention_Type.featOkTst && ((CCPClassMention_Type)jcasType).casFeat_slotMentions == null)
069          jcasType.jcas.throwFeatMissing("slotMentions", "edu.uchsc.ccp.uima.mention.CCPClassMention");
070        jcasType.ll_cas.ll_setRefValue(addr, ((CCPClassMention_Type)jcasType).casFeatCode_slotMentions, jcasType.ll_cas.ll_getFSRef(v));}    
071        
072      /** indexed getter for slotMentions - gets an indexed value - A class mention optionally has slot mentions which represent attributes of that class. These slot mentions are stored in the slotMentions FSArray. There are two types of slot mentions, complex and non-complex.  The difference between complex and non-complex slot mentions is simply the type of filler (or slot value) for each. Complex slot mentions are filled with a class mention, whereas non-complex slot mentions are filled by simple Strings.
073       * @generated */
074      public CCPSlotMention getSlotMentions(int i) {
075        if (CCPClassMention_Type.featOkTst && ((CCPClassMention_Type)jcasType).casFeat_slotMentions == null)
076          jcasType.jcas.throwFeatMissing("slotMentions", "edu.uchsc.ccp.uima.mention.CCPClassMention");
077        jcasType.jcas.checkArrayBounds(jcasType.ll_cas.ll_getRefValue(addr, ((CCPClassMention_Type)jcasType).casFeatCode_slotMentions), i);
078        return (CCPSlotMention)(jcasType.ll_cas.ll_getFSForRef(jcasType.ll_cas.ll_getRefArrayValue(jcasType.ll_cas.ll_getRefValue(addr, ((CCPClassMention_Type)jcasType).casFeatCode_slotMentions), i)));}
079    
080      /** indexed setter for slotMentions - sets an indexed value - A class mention optionally has slot mentions which represent attributes of that class. These slot mentions are stored in the slotMentions FSArray. There are two types of slot mentions, complex and non-complex.  The difference between complex and non-complex slot mentions is simply the type of filler (or slot value) for each. Complex slot mentions are filled with a class mention, whereas non-complex slot mentions are filled by simple Strings.
081       * @generated */
082      public void setSlotMentions(int i, CCPSlotMention v) { 
083        if (CCPClassMention_Type.featOkTst && ((CCPClassMention_Type)jcasType).casFeat_slotMentions == null)
084          jcasType.jcas.throwFeatMissing("slotMentions", "edu.uchsc.ccp.uima.mention.CCPClassMention");
085        jcasType.jcas.checkArrayBounds(jcasType.ll_cas.ll_getRefValue(addr, ((CCPClassMention_Type)jcasType).casFeatCode_slotMentions), i);
086        jcasType.ll_cas.ll_setRefArrayValue(jcasType.ll_cas.ll_getRefValue(addr, ((CCPClassMention_Type)jcasType).casFeatCode_slotMentions), i, jcasType.ll_cas.ll_getFSRef(v));}
087       
088        
089      //*--------------*
090      //* Feature: ccpTextAnnotations
091    
092      /** getter for ccpTextAnnotations - gets Just as CCPTextAnnotations are linked to a CCPClassMention, it is sometimes useful to be able to follow a CCPClassMention back to its corresponding CCPTextAnnotations, therefore, this FSArray contains links to the CCPTextAnotation(s) for this class. NOTE: The use of an FSArray is probably not needed here as there is typically only one occupant in the FSArray.
093       * @generated */
094      public FSArray getCcpTextAnnotations() {
095        if (CCPClassMention_Type.featOkTst && ((CCPClassMention_Type)jcasType).casFeat_ccpTextAnnotations == null)
096          jcasType.jcas.throwFeatMissing("ccpTextAnnotations", "edu.uchsc.ccp.uima.mention.CCPClassMention");
097        return (FSArray)(jcasType.ll_cas.ll_getFSForRef(jcasType.ll_cas.ll_getRefValue(addr, ((CCPClassMention_Type)jcasType).casFeatCode_ccpTextAnnotations)));}
098        
099      /** setter for ccpTextAnnotations - sets Just as CCPTextAnnotations are linked to a CCPClassMention, it is sometimes useful to be able to follow a CCPClassMention back to its corresponding CCPTextAnnotations, therefore, this FSArray contains links to the CCPTextAnotation(s) for this class. NOTE: The use of an FSArray is probably not needed here as there is typically only one occupant in the FSArray. 
100       * @generated */
101      public void setCcpTextAnnotations(FSArray v) {
102        if (CCPClassMention_Type.featOkTst && ((CCPClassMention_Type)jcasType).casFeat_ccpTextAnnotations == null)
103          jcasType.jcas.throwFeatMissing("ccpTextAnnotations", "edu.uchsc.ccp.uima.mention.CCPClassMention");
104        jcasType.ll_cas.ll_setRefValue(addr, ((CCPClassMention_Type)jcasType).casFeatCode_ccpTextAnnotations, jcasType.ll_cas.ll_getFSRef(v));}    
105        
106      /** indexed getter for ccpTextAnnotations - gets an indexed value - Just as CCPTextAnnotations are linked to a CCPClassMention, it is sometimes useful to be able to follow a CCPClassMention back to its corresponding CCPTextAnnotations, therefore, this FSArray contains links to the CCPTextAnotation(s) for this class. NOTE: The use of an FSArray is probably not needed here as there is typically only one occupant in the FSArray.
107       * @generated */
108      public CCPTextAnnotation getCcpTextAnnotations(int i) {
109        if (CCPClassMention_Type.featOkTst && ((CCPClassMention_Type)jcasType).casFeat_ccpTextAnnotations == null)
110          jcasType.jcas.throwFeatMissing("ccpTextAnnotations", "edu.uchsc.ccp.uima.mention.CCPClassMention");
111        jcasType.jcas.checkArrayBounds(jcasType.ll_cas.ll_getRefValue(addr, ((CCPClassMention_Type)jcasType).casFeatCode_ccpTextAnnotations), i);
112        return (CCPTextAnnotation)(jcasType.ll_cas.ll_getFSForRef(jcasType.ll_cas.ll_getRefArrayValue(jcasType.ll_cas.ll_getRefValue(addr, ((CCPClassMention_Type)jcasType).casFeatCode_ccpTextAnnotations), i)));}
113    
114      /** indexed setter for ccpTextAnnotations - sets an indexed value - Just as CCPTextAnnotations are linked to a CCPClassMention, it is sometimes useful to be able to follow a CCPClassMention back to its corresponding CCPTextAnnotations, therefore, this FSArray contains links to the CCPTextAnotation(s) for this class. NOTE: The use of an FSArray is probably not needed here as there is typically only one occupant in the FSArray.
115       * @generated */
116      public void setCcpTextAnnotations(int i, CCPTextAnnotation v) { 
117        if (CCPClassMention_Type.featOkTst && ((CCPClassMention_Type)jcasType).casFeat_ccpTextAnnotations == null)
118          jcasType.jcas.throwFeatMissing("ccpTextAnnotations", "edu.uchsc.ccp.uima.mention.CCPClassMention");
119        jcasType.jcas.checkArrayBounds(jcasType.ll_cas.ll_getRefValue(addr, ((CCPClassMention_Type)jcasType).casFeatCode_ccpTextAnnotations), i);
120        jcasType.ll_cas.ll_setRefArrayValue(jcasType.ll_cas.ll_getRefValue(addr, ((CCPClassMention_Type)jcasType).casFeatCode_ccpTextAnnotations), i, jcasType.ll_cas.ll_getFSRef(v));}
121      }
122    
123
```
